# Ableism in Applied Behavior Analysis: A Beginner’s Guide to Understanding and Dismantling Ableism in Practice with Autistic People

**DOI:** 10.1007/s40617-025-01128-y

**Published:** 2025-12-10

**Authors:** Jennifer J. McComas, Susan Wilczynski, Mari-Luci Cerda, Hannah S. Beavis, Claudia Drossel, Shawnna Sundberg, Kaston D. Anderson

**Affiliations:** 1 Department of Educational Psychology, University of Minnesota, Minneapolis, MN USA; 2 Ball State University, Muncie, IN USA; 3 Lighthouse of Equitable Access and Practices (LEAP), Lubbock, TX USA; 4 Eastern Michigan University, Ypsilanti, MI USA; 5 Department of Psychology, Michigan State University, East Lansing, MI USA

**Keywords:** Ableism, Applied behavior analysis, Behavior analysis, Autism spectrum disorder, Neurodiversity, Neurodivergent, Bias, Ethics, Private events

## Abstract

Ableism is biased covert behavior or overtly discriminatory actions against people who are disabled. Ableism often involves words and actions that convey an attitude or belief that disabled people are inferior to nondisabled people, irrespective of whether the person holding these views (i.e., private events) is aware that their thoughts are biased or actions are discriminatory. All people have biases that have grown out of their exposure to harmful social norms, models, and observational learning as well as experiences and contingencies. As practitioners, administrators, instructors, and scientists, we are responsible for recognizing our own biases and actively working to alter our words and behavior so that our biases do not manifest in discrimination. This paper describes ableism and its impact on applied behavior analytic (ABA) practices, services, and supports and on the Autistic people we serve. The authors provide a brief discussion of the current efforts to reform ABA services and where they view anti-ableism is situated in the reform ABA movement. Then the bulk of the paper features examples of ableist practices and suggestions for dismantling ableism in ABA practice. Although these suggestions largely have not yet been submitted to empirical investigation, the general paucity of research in this area combined with the ethical directive to avoid discrimination requires practitioners to begin this work while empirical research is in its infancy. The companion article “Ableism in Applied Behavior Analysis: Historical Context of Services for Autistic People” (McComas et al., in press) provides a more in-depth exploration of the historical context of ableism in ABA.

## What is Ableism

In an early reference to the contemporary notion of ableism, rehabilitation psychology scholar and disability advocate Lee Myerson^1^ pointed out that social and psychological problems rather than physical impairments are the major problem of the “handicapped” (Meyerson, 1948). Ableism involves biased thoughts (i.e., private events) and discriminatory behaviors against disabled people. Thoughts, beliefs, and practices that do not consider the perspectives of disabled people, those with intellectual disabilities, or those with mental health needs are inherently, if unintentionally, ableist. The natural consequences of this lack of regard include marginalizing and making disabled people feeling “othered” (Chouinard, 1997). Ableism dehumanizes people who are disabled (Campbell, 2001), and to Meyerson’s point (1948), instead of recognizing the role of the social environment, squarely lays blame on the disabled person and “their impairment” (Amundson & Taira, 2005). In fact, different does not mean bad (Gernsbacher & Yergeau, 2019). Much like biodiversity is necessary for successful environments (Walker, 2021), we should acknowledge that diversity in neurological functioning is valued and necessary for successful societies. Accordingly, it is essential that our scientist-practitioner lens does not obscure our ability and responsibility to recognize the strengths, capabilities, and meaningful contributions of Neurodivergent and Autistic people (Black et al., 2024; Rentenbach et al., 2017). As fellow humans, their perspectives merit our attention, respect and inclusion in influencing practices that affect their lives (Dunn, 2022).

Discrimination can be explicit (blatant and purposeful actions; Keilman, 2024) or implicit (often unintentional but still causing harm; Friedman, 2019). Whether discrimination is implicit or explicit, practitioners who engage in discrimination compromise equity of care (VanPuymbrouck et al., 2020) with regard to access, comprehensiveness, and quality. On the basis of our experience with board-certified behavior analysts (BCBAs), we operate on the assumption that practitioners seek to provide high-quality services. These services may also include unintentional discrimination in one or more of the forms we describe below. We restrict our focus in this article to implicit discrimination and bias that Autistic^2^ people have experienced when receiving applied behavior analytic (ABA)-based services, based on practitioner behaviors observed by the authors in ABA-based service settings and those reported by Autistic people who have received ABA-based services (see Anderson, 2023). We acknowledge that these observations and reports are a fraction of and may not be representative of practitioners’ behavior more broadly. However, the impact of even a fraction of the services we provide involving ableism and discrimination, albeit unintentional, warrants our concern.

Although there is growing discourse among behavior analysts and researchers regarding the dismantling of ableism and a greater emphasis on assent (Breaux & Smith, 2023) and compassionate care (Taylor et al., 2019), the body of empirical research supporting these developments in applied behavior analysis (ABA) remains in its early stages. Indeed, scholars and advocates alike have issued calls for reforming ABA in one way or another. Emphasis on socially valid practice in ABA (Callahan et al., 2017; Wilczynski, 2024; Wolf, 1978) has evolved to include calls for reform, conceptually anti-ableist approaches, or both (Johnson, 2025) and have articulated the need for compassionate care (e.g., Denegri et al., 2023; Rodriguez et al., 2023), assent-based services (Flowers & Dawes, 2023; Morris et al., 2024), neurodiversity affirming care (Allen et al., 2024; Mathur et al., 2024), and trauma-informed care (Rajaraman et al., 2022). We believe these calls are all connected to the distinct but related manifestations of ableism that include bias, discrimination, and even stigma^3^ (see Čolić, 2025). A comprehensive discussion of dismantling bias, discrimination, and stigma in ABA is beyond the scope of this paper; in fact, doing so would likely require an entire book. Nonetheless, our aim is to highlight the unifying thread of ableism and offer actionable recommendations for reflection and reform (i.e., reflective practice; Cirincione-Ulezi, 2020; Taylor et al., 2024).

## How does Ableism Show Up in Applied Behavior Analysis?

### Bias

Bias can take several forms, including systemic/structural, interpersonal explicit, and interpersonal implicit and can be operationally defined as differential responding toward members or groups based on characteristics that do not warrant such variation (Wray et al., 2009). Systemic/structural bias is characterized by routine institutional policies, practices, or procedures, embedded in the operating systems of an organization or society, that create advantages for certain groups and disadvantages for others. In the field of ABA, examples might include insurance approvals that encourage behavior analysts to pathologize autism in order to approve reimbursement (Johnson, 2025) and insurance companies requiring a diagnosis of autism to approve ABA services as medically necessary, despite the fact that other clients could benefit from ABA services. Interpersonal bias involves unfair or unjust perceptions or judgements by one person about another. Although interpersonal bias can be explicit, our focus is on the implicit. Implicit bias occurs when people are unaware of their covert verbal behavior (i.e., attitudes, thoughts) that stereotype members of a group based on their group membership (De Houwer, 2019). Implicit bias and discrimination (Dickter et al., 2020; Friedman, 2019) warrant careful consideration and behavior change because Autistic people can experience harm even when practitioners are very well-intentioned. Ableist biases, like other biases, are a result of the person’s culturally mediated learning histories (Wilczynski, 2024).

Examples of differential responding connected to implicit biases include when practitioners interact with Autistic people in a paternalistic manner or make condescending statements (Dickter et al., 2020; Jaramillo & Nohelty, 2022; Nario-Redmond et al., 2019). A practitioner’s implicit bias may manifest in different observable behaviors with different Autistic people. For example, practitioners may make decisions for Autistic people without regard for their personal priorities and preferences; they may disregard their perspectives and strengths, downplay their support needs for building particular kinds of skill sets, or not adequately address their mental health needs (Botha & Frost, 2020). Practitioners may limit the person’s self-determination (Peterson et al., 2021) and de-emphasize social validity in professional decision-making because they are responding to implicit biases they have adopted from the larger culture and the culture of their field (Wilczynski, 2024). Implicit bias regarding Autistic people who have co-occurring diagnoses of communication disorders or intellectual disabilities might further involve assuming they are not capable of having meaningful preferences or priorities, interpreting behavior outbursts solely as problem behavior to be reduced instead of considering the communicative function of the outburst (e.g., identifying relevant motivating operations or aversive stimulus conditions in the environment), and infantilization or dehumanization (e.g., ignoring the need for sexuality education; Campbell et al., 2020; Herrick & Datti, 2022).

### Selection of Behavior/Skill Targets, Procedures, and Outcomes

Socially valid practice requires that behavior analysts consider the degree to which the goals (behavior/skill targets), procedures, and outcomes of instruction and intervention are socially important, acceptable, and meaningful to the individual receiving services, their caregivers, and the broader community (Huntington et al., 2024; Wolf, 1978). In anti-ableist terms, we must be critical of intervention goals that include eradication of behavior that is not “normal” (Veneziano & Shea, 2023). Discrepancies between what neuro-majority and Autistic people perceive as desirable behavior or skill targets, interventions, and outcomes can stem from behavior analysts’ implicit biases, particularly when authentic evidence of social validity is not procured. When behavior analysts plan and implement services for Autistic people, they typically rely on assessment results, their training, experience, and evidence-based practices. Often, behavior analysts interview interested parties (e.g., parents or other caregivers) to select behavior/skill targets, intervention procedures, and outcomes. Less commonly do they identify the Autistic person’s perspective regarding which responses to target, which interventions are preferred, or outcomes are valued (Beaulieu et al., 2019). To clarify, we are not suggesting that practitioners expect Autistic individuals with intellectual disabilities, high support needs, and minimal communication skills to verbally articulate their perspectives on each dimension of social validity. Nonetheless, it is important to recognize that social validity remains a relevant consideration irrespective of the Autistic person’s relative strengths or support needs. For example, preference assessments (Hanley, 2010), concurrent chain procedures (Fulton et al., 2020), and observing and respecting indications of assent and assent withdrawal (Breaux & Smith, 2023) must be fully utilized to incorporate the perspective of the Autistic person receiving behavioral services into the intervention planning and implementation process (Johnson, 2025; Tullis & Carnett, 2025; Wilczynski, 2024).

Behavior/skill targets related to preparing children for inclusive educational settings risk placing the onus of success and “fitting in” on the disabled child, aiming for them to conform to neuro-majority expectations about how to “be” or “act” (Johnson, 2025). We acknowledge that some behaviors are non-negotiable. For example, Autistic school children cannot be permitted to physically hurt others based on the rationale that “this is part of their autism.” However, behavior analysts should challenge their assumptions before recommending specific targeted responses or skills and make goal selection linked to a careful consideration of the guiding principles and ethical codes (Behavior Analyst Certification Board, 2022). In addition, behavior analysts can aid educators in teaching all students to be kind to others in various situations, embrace differences (including their own), and include peers from the “outgroup.^4^” Indeed, as Schuck et al., (2022; p. 1) point out, “there is a general consensus that the unique attributes associated with autism and other neurocognitive disabilities should not be distilled down to a set of symptoms and vulnerabilities in need of correction. Instead, neurodiversity perspectives assert a need to promote societal education, acceptance, and accommodation of the neurocognitive differences of these individuals.”

Autistic people may receive interventions to decrease or replace responses that often are considered a problem only by the neuro-majority. To illustrate, when a disabled person engages in repetitive actions or utterances, some behavior analysts consider these behavior patterns stigmatizing and therefore target them for elimination, yet research has shown that repetitive behavior (not deemed dangerous to the self or others) is beneficial for both self-regulation and communication (Cherewick & Matergia, 2024; Martínez-González et al., 2022). Examples of repetitive behavior patterns that rarely need to be decreased are body-rocking, hand flapping (referred to by many in the autism community as stimming), and talking to oneself (Morris et al., 2025). Historically, stimming behaviors have been targeted for decrease to “help” the Autistic person appear more “normal” with the sincere hope they would not face bias or stigma and instead, they would “fit in” socially. Yet, those aims were identified by interested parties (e.g., parents, teachers, clinicians) who overlooked the fact that these responses may involve regulation (McCormack et al., 2023) and sensory needs (Cherewick & Matergia, 2024), aversive conditions such as hyperacusis (Danesh et al., 2021), or simply a unique way of being (Späth & Jongsma, 2020). Experiencing procedures designed to reduce responses that are adaptive for an Autistic person have been reported to lead to adverse situations such as increased tantrums, social withdrawal, dysregulation, masking, and emotional harm to Autistic people (see Cage & Troxell-Whitman, 2019; Cage et al., 2018; Ne’eman, 2021). People who access behavioral health services have a right to effective service that is culturally responsive, socially valid, and centers individual preferences (see McVey et al., 2023). The ableist practice of targeting for reduction behaviors that serve a self-regulatory function within behavior analysis and other fields is inconsistent with these rights. Thus, behavior analysts can inadvertently cause harm (see Torrado et al., 2017) and violate Autistic people’s rights as well as their own ethical responsibilities, despite the best of intentions. Despite the ethical requirement for applied behavior analysts to involve their clients and interested parties in selecting goals and interventions (Behavior Analyst Certification Board, 2022), still too prevalent is the practice of following prevailing societal expectations to decrease behavior such as stimming. Many service providers have pursued improved collaboration with clients to identify goals, procedures, and outcomes of their services. These behavior analysts are a model for others who could improve their approach to shared decision-making with their clients and decrease their focus on behaviors like stimming.

Ableist thoughts or actions can influence the many decisions that practitioners make regarding goals. Prioritizing the topographical response without considering context and failing to bring behavior under relevant stimulus control (i.e., neglecting to teach the Autistic person to discriminate the contexts in which some responses are and are not appropriate) are examples of where ableism might show up in behavior analytic practice. Examples of behaviors that might be context-dependent could include eye contact (or looking elsewhere when someone is talking to you), sexual behavior, and refusing to engage in a non-preferred activity. Members of the neuro-majority often do not make eye contact with social partners when they are deep in thought or experiencing anxiety (Argyle & Dean, 1965; Clin & Kissine, 2023). When similar responses by Autistic people are pathologized, the double standard should be called into question. Similarly, neuro-majority adults do not find it problematic to leave work or social settings in which they are not enjoying themselves but presume Autistic people should learn to “tolerate” these conditions (e.g., Powers & Thorwarth, 1985). Ableism is the most likely explanation when the criterion for what is considered “acceptable” behavior is different for the neuro-majority than it is for Autistic people because context is considered relevant for the former but not the latter group. In other words, expecting an Autistic person to tolerate social adversity without offering the same understanding or flexibility extended to the neuro-majority can reflect an ableist perspective.

We argue that behavior analysts who implement socially valid and anti-ableist interventions provide the instruction and support needed for Autistic people to exercise their rights to assent and to withdraw assent unless there is an overwhelmingly compelling reason to do otherwise. The goal should be to obtain assent and, to the maximum extent possible, to amplify self-determination and enhance choice throughout all behavioral service provision for Autistic people (Morris et al., 2021). Preferences for instructional approaches and interventions can be expressed vocally or in other ways (enthusiasm, negative affect, disengagement, refusal). This is not to suggest individual preferences should be prioritized when safety might be compromised or long-term quality of life can be harmed (Taylor et al., 2023), but rather, to encourage practitioners to challenge their own assumptions before concluding that honoring preferences, values, and self-determination is unnecessary or less important. A common concern expressed about assent-based care is the potential for lost instructional time when the Autistic person withdraws assent. This concern is valid and warrants thoughtful attention. Still, it remains essential for behavior analysts to identify factors that enable assent (e.g., changing stimulus materials, modifying the timing or sequence of tasks). In addition, unnecessarily requiring an Autistic person to continue to “tolerate” conditions they find aversive may inadvertently contribute to an increase in behavior that interferes with learning (e.g., increased stereotypic behavior, meltdowns, aggression). Further, learning will be compromised when a person’s autonomic system is hyper-aroused (Vogel & Schwabe, 2016). Ironically, this means the goal of increasing instructional time by requiring Autistic people to persevere under these suboptimal conditions may actually decrease access to effective instructional time.

In terms of outcomes, practitioners who rely solely on graphed results of target behaviors/skills can only establish whether a goal has been met; they cannot evaluate whether meaningful outcomes have been achieved. Alternately, including feedback about how the quality of life has been improved, the restrictiveness of the environment has been reduced, and opportunities have been expanded (Gitimoghaddam et al., 2022; Schwartz & Kelly, 2021) is more socially valid and less ableist (Wilczynski, 2024) and allows an evaluation of meaningful outcomes. The insufficient attention in the literature to all three dimensions of social validity may have increased the risk of ableism in practice. When researchers set the research agenda without input from interested parties (Pritchett et al., 2022; Roche et al., 2021), they may be inadvertently modeling the belief that experts are most qualified to identify responses to be targeted, interventions to be implemented, and outcomes to be achieved and consultation from Autistic people is unnecessary.

The BACB ethics code (Behavior Analyst Certification Board, 2022) requires a vested commitment to honoring disabled people’s perspectives and lived experiences (i.e., culturally mediated learning histories) to guide the development of effective behavioral interventions with social validity. Sections 2.09 (Involving Clients and Stakeholders) and 2.14 (Selecting, Designing, and Implementing Behavior-Change Interventions) emphasize the importance of supporting client agency in behavioral interventions. For Autistic people, this includes honoring their lived experiences and conducting culturally responsive assessments and interventions (El-Ghoroury et al., 2012; Fong, 2020; Jimenez-Gomez & Beaulieu, 2025; Johnson, 2025; La Roche et al., 2018). Furthermore, Board Certified Behavior Analysts (BCBAs) must examine the environmental conditions that may interfere with or affect intervention implementation and the appropriateness of all planned programs (Sect. 2.19). To extend the language of the Ethics Code, behavior analysts should pay particular attention to private events (e.g., implicit biases, prejudices, and stereotypes) that influence their intervention and program development. By considering their own private setting events and incorporating the learner’s preferences and priorities in planning behavioral programs, the behavior analyst can facilitate Autistic people’s sense of ownership of the behavioral programs and exemplify the BACB Code of Ethics ethos. Ethical codes sometimes conflict with each other, and behavior analysts will want to use their guiding principles to help them resolve these conflicts in a way that serves the Autistic person’s best interest (Wilczynski, 2024).

### Written and Oral Communication

Through language, we construct our identities, express our beliefs, and either support or marginalize people within or outside of our communities. Using certain language can convey and perpetuate ideologies about Autistic people, which may significantly impact their lives (Bottema-Beutel et al., 2024). Behavior analysts describe Autistic people when they write research reports as well as individualized instruction and support plans. For individualized instruction and support plans, behavior analysts specify the person’s present level of performance, goals, and objectives in quantifiable and time-specified ways. A critical examination of reports and plans may reveal numerous examples of ableism. To begin, documents may contain descriptions of individual differences as “challenges” and terms referencing the individual as “maladaptive,” “antisocial,” or “low-functioning” (Monk et al., 2022), particularly compared to neuro-majority norms. In addition, behavior analysts, like professionals in many other disciplines, reference antiquated research that pathologizes differences (e.g., particular play and leisure preferences or stimming behavior; Kapp et al., 2019) and use terminology that does not adequately capture Autistic people’s experiences (Bottema-Beutel et al., 2024) rather than seeking and conveying a Neurodivergent perspective (Amaral, 2023).

Behavior analysts are responsible for preparing individualized instructional and support plans that identify individual needs, strengths, and optimal learning conditions that guide practitioners in facilitating skill development in the area of self-determination toward advocacy of their own goals, interventions, and outcomes (Wilczynski, 2024). That is, the nuanced messages conveyed within documents generated by behavior analysts can either enhance or undermine the likelihood that social validity and cultural sensitivity can be achieved. Whether in conversation or writing, behavior analysts can typically describe differences and disabilities in a manner that communicates the need for services while upholding human dignity and actively avoiding depicting differences or people as inferior. For example, behavior analysts can acknowledge the difficulty in managing meltdowns to respect the parents’ experience, and they can also recognize this as a rudimentary form of self-advocacy, which would benefit from skill development so that the Autistic child’s needs can be more efficiently met. Similarly, it may be useful to convey that stereotypic behavior often occurs when an Autistic adult is under distress and that these behaviors are highly adaptive. Further, this example represents an excellent opportunity to clarify that the behavior analyst will complete an assessment of motivating operations so that the environment can be made less aversive or so that the Autistic adult can complete their work in an alternative setting where the aversive conditions are minimized. Describing differences in a way that upholds human dignity more clearly comports with laws and ethical codes that require person-centered involvement in decisions about their own lives and that avoid unnecessary harm by ensuring people view themselves as skilled, valued, worthy of support, and confident that the record accurately depicts their differences, strengths, and support needs without implicit comparison to neuro-majority people. Altering the way behavior analysts communicate with and about Autistic people requires a re-examination of our assumptions in order to communicate in a more effective, non-ableist way.

## Action Steps for Dismantling Ableism in Applied Behavior Analysis

The start of dismantling any form of discrimination is to become aware of our biased thinking and how this biased thinking might unintentionally influence discrimination in our practice (Beaulieu & Jimenez-Gomez, 2022). Our approach to spotlighting ableism, reflecting on our own practices, and advocating for the people we work with has evolved with our roles as ethically driven scientist-practitioners. We draw heavily from the work of other scholars focusing on disability critical race theory (Annamma et al., 2012). Understanding the power imbalance and structural systems that maintain ableism allows us to generalize and apply research findings on systemic racism to systemic ableism.

Ethics training in applied behavior analysis must not only address the potential of the science of human behavior to effect meaningful change but also emphasize that behavior analysts are obligated to consider the individual’s context in which change techniques will be implemented (Ming et al., 2023). Behavior analytic efforts to improve individual and family quality of life require careful examination of the Autistic person positioned in their sociocultural context (BACB ethics Code 1.07, Cultural Responsiveness and Diversity). All too often, this context includes bias and discrimination, including practitioners who intend to benefit their Autistic learners but are not aware of the harm their ableist practices may cause (Wilczynski, 2024).

Dismantling ableism is long overdue; the time for action is now. Discourse about affirming neurodiversity in ABA has begun (Allen et al., 2024; Graber & Graber, 2023; Mathur et al., 2024; Rodriguez et al., 2023; Shyman, 2016), and awareness of Autistic and Neurodivergent perspectives is growing (Botha & Cage, 2022; Chazin et al., 2024; see Johnson, 2025). Skilled in the application of behavioral principles, we have the tools to change ableist behavior (e.g., Nario-Redmond, 2019). In this context, behavior change requires constantly revisiting issues of ableism, bias, and discrimination and being part of the solution regarding inclusion and acceptance of autism, neurodiversity, and, more broadly, disability. The same methods we apply to habit reversal can be applied to reducing bias and discrimination (Forscher et al., 2017), which begins with increasing awareness of our ableist thoughts and actions.

As scientist-informed practitioners who aspire to center the priorities and perspectives of those we serve, we recognize that both of the following are true: ABA-based interventions and supports are effective, and Autistic people have described the same interventions and supports as ableist and not socially valid, therefore harmful (Anderson, 2023; Johnson, 2025). We can retain the application of effective behavioral principles in a socially valid way by actively ensuring we involve Autistic people in program planning, implementation, and evaluation and by dismantling ableism in our practice. Involving Autistic people begins with assessing how to support them best to participate in these processes given their current skills and identifying it as our responsibility to help them acquire the skills needed to participate in and, ideally, eventually lead these efforts whenever possible. Dismantling ableism in practice requires both reflection (verbal behavior, private events) and action (overt behavior). Because ableism is ubiquitous throughout the larger culture in which we live (Reber et al., 2022) and work, meaningful and intentional actions will need to be made at both the individual (e.g., honoring the preference many Autistics have for identity-first language or assessing preference for which instructional targets to address) and systemic levels (e.g., altering our decision-making process regarding what is an appropriate target behavior).

As we work to continuously self-assess and alter our own behavior, we will likely need to become accustomed to engaging other behavior analysts by using tactics like *calling in* and, in some situations, *calling out* (Harvard Diversity Inclusion and Belonging, n.d.). Calling in and calling out both involve bringing ableist behavior, including biased views and discriminatory actions, to the attention (awareness; Skinner, 1957) of the person who makes statements or takes actions reflecting bias and/or discrimination. Calling out involves publicly confronting bias/discrimination as it occurs even if others are present, because the consequence of failing to do so may be harmful. Being called out is often experienced as aversive, which reduces the likelihood both parties can benefit from the exchange of views in a way that results in less ableist service delivery. Thus, calling in is the preferred method to use unless it is unavoidable. Calling in involves having a private conversation with the person to discuss your perception that their behavior may be ableist and the associated implications of their actions. Although learning that your actions may be ableist in nature may be experienced as aversive, it is likely to be far less aversive than being called out in front of other people because the social punishment may be greater in a public context.

The context and the ramifications of calling in or calling out should be considered before either action is considered. For example, a panelist making ableist statements at a conference may have a systemic negative impact on service delivery when a large audience deems the ableist statements as acceptable because a respected, knowledgeable person with several degrees and years of experience in the field delivered them. Before proceeding, the moderator must consider the risks associated with calling in, calling out, or inaction. Waiting until the presentation is completed to call in the panelist risks the audience being unaware that an ableist statement was made and the audience members may be likely to make similar statements due to observational learning. Calling out the panelist may help the audience understand that the statement is ableist but there is also a risk of audience members simply labeling the speaker as a “bad person” rather than using it as an opportunity to reflect on their own ableist thoughts or actions. Inaction means no one benefits from the insight that an ableist statement has been made and that the current level of ableist views among audience members will remain the same or increase. In this case, inaction is the worst option. In addition to the moderator, the other panelists and the discussant will need to weigh the best way to proceed.

In an extension of this example, consider when an Autistic panelist (Panelist 1) states, “My experience with ABA-based services was traumatic. I don’t recall ever giving assent and although it was helpful for changing my highly destructive behavior, it could have been implemented more compassionately so that it felt more supportive than aversive” and Panelist 2 who is a professor, scholar and practitioner in ABA with over a decade of experience counters with, “As a scholar of ABA, I can tell you that ABA is not abusive and does not cause trauma.” The moderator could use this opportunity to call out Panelist 2’s ableist statement by saying something like, “We are all learning how to discuss issues of ableism in applied behavior analysis, and I would like to take a moment to think aloud about the differences in what has been said by Panelist 1 and Panelist 2. Panelist 1 has experience as a recipient of ABA services and shared that their experience was traumatic, even though it also had associated benefits. Panelist 2 suggested that ABA is not abusive and does not cause trauma. How do we resolve these different views? First, it is essential to carefully consider and genuinely engage with the perspectives of individuals who have received ABA services. Panelist 1’s comments are similar to those that have been popularized on social media, and these statements suggest a lack of social validity—at least some of the time, for some clients, students, or consumers. Additionally it is important to acknowledge that we have not consistently or comprehensively researched adverse effects of ABA. We cannot reasonably draw the conclusion that ABA is not abusive or that it does not lead to trauma if we do not have the data to support that contention. We need researchers to collect these kinds of data. As we reflect together, I’d like to invite all of us—both panelists and audience members—to consider the importance of this different perspective. Panelist 1, if you feel comfortable sharing, would you be willing to elaborate on the specific procedures and how they were implemented that led you to feel this way or the conditions under which you might have assented to a particular procedure? Gaining some perspective into this could help behavior analysts to consider modifications or alternative tactics to the behavior analytic procedure that might have both desirable effects on target behavior and respect the dignity and autonomy of the individual receiving services.” In this case, the moderator calling out Panelist 2 gives the audience an opportunity to gain a deeper understanding than a binary good/bad judgement about either ABA-based practices or the panelists that could lead to ostracization. As a member of the presentation, Panelist 2 is welcome to add their response without invitation.

Dismantling ableism in the practice of ABA is possible when behavior analysts use their knowledge of behavioral principles to alter contingencies for themselves (e.g., self-management via habit reversal) and others, to truly consider individual preferences, to target skills that will facilitate autonomy and self-determination, and to revise the nature of the relationship between practitioners and disabled people (e.g., social validity based on the Autistic person’s point of view; Johnson, 2025).

To produce the large-scale socially meaningful changes needed in our field as well as the larger culture in which we live, we suggest considering ways practitioners might address ableism. Table 1 provides some examples of how anti-ableist practitioners can produce socially meaningful changes for themselves, for ABA-service users, and our communities. The table provides many examples of suggestions for altering ableist behavior. We fully acknowledge that many of these behavior patterns we refer to as ableist can have multiple explanations and that the anti-ableist approaches must be individualized for people with a range of support needs. Nonetheless, we hope that these examples provide a starting place for beginning to dismantle ableism that is detected when we engage in deep reflection and commitment to change. Clearly, we cannot eliminate all ableist behavior patterns in our repertoire simultaneously and immediately. In addition, behavior analysts must avoid mindlessly forbidding some applications of behavioral principles without due consideration to the ethical risks an individual may experience if we fail to provide supports that best improve their quality of life in the long-term (Delgado et al., 2023; Taylor et al., 2023). Yet, we can prioritize dismantling ableist behaviors in our own practice, with consideration for both what actions are manageable for behavior analysts and important to Autistic people. Although immediately dismantling all ableism from our practice when we become aware of it is unlikely, we can work to individually and systematically apply the power of behavior analysis technology to change and improve our practice and field.

**Table 1:** Examples of current practices with considerations to target alternative practices

Topic	If this describes your current practice	Then consider targeting this change in practice
Working with Autistic People	• Using person-first or identity-first language for all people (note: The service user should make this determination) • Using ableist language choices when talking to and about learners • Immediately programming and designing goals to promote “fitting in” or acting a certain way	• Accept Autistic people as they are, respecting their preferences and self-determination • Collaborate with and seek input from the Autistic community • Ask people their preferences for person-first or identity-first language (Pittsburgh Center for Autistic Advocacy, 2018) • Determine whether the individual requires help before doing a task or answering a question for them • Use current anti-ableist terminology (e.g., replace “at risk for autism” with “at elevated likelihood,” replace “comorbid” with “co-occurring” and inclusion of specific descriptors of functioning or support needs, Natri et al., 2023) For additional examples see the American Psychological Association’s bias-free and inclusive language guidelines (APA, 2021) • Check own assumptions before recommending behaviors or skills to be targeted • Endeavour for ABA training to include instruction on components related to sleep, mental health, developmental psychology, genetics, eating and their relation to disability status (Dreiling et al., 2022; Luiselli, 2021)
Assessment	• Neglecting to collect and/or use data on motivating operations that can lead to variability in performance • Using neuro-majority performance as the normative criteria for social behavior • Conducting preference assessments to arrange contingencies but not to identify the Autistic individual’s preference for instructional programs	• Collect data on common motivating operations (e.g., sleep, hunger, etc.) as well as idiosyncratic motivating operations unique to each person to inform daily intervention decisions • Seek input from the Autistic community regarding performance criteria for social behavior • Conduct assessments to identify preference for instructional goals/programs through a trauma informed lens (Rajaraman et al., 2022) • Include assent and consent processes during all phases of assessment (for discussion on assent see Breaux & Smith, 2023)
Selecting Service Goals	• Setting “curing” or recovery from autism as the goal • Setting goals that aim to change Autistic people so they behave more “like” everyone else • Targeting stereotypic responses for reduction independent of context • Setting a standard for expected behaviors higher than the general population (e.g., 100% compliance with instructions) • Failing to provide comprehensive sexuality education • Ignoring or suppressing anger or frustration • Interpreting resistance as “problem behavior” instead of preference for a change in the current environmental arrangement • Designing and implementing services that are interchangeable across people	• Set goals based on self-determination, particularly as people leave early childhood (see Anderson, 2023 for discussion on self-determination) • Use Motivational Interviewing when possible to identify goals (Frielink & Embregts, 2013) • Provide person-centered care and apply collaborative planning strategies • View stereotypic responses (that are not dangerous to self/others) as an opportunity to (a) accept the behavior, (b) conduct a structural (antecedent) analysis to understand and make necessary adjustments to the environment, or both (Cooper et al., 1992) • Educate professionals/caregivers to be critical consumers of goals, particularly when referral goals reflect “looking normal” or performing a response at the same rate as neuro-majority people • Collaborate with qualified sexuality educators to address intimate and sexual relationship behaviors across the lifespan, including developing awareness and advocacy for sensory differences related to sex (aversions and needs) (see Herrick & Datti, 2022 for a review) • View upset, anger, and/or frustration as a common human experience and respond accordingly with compassion. Equally, view these as a signal for the need to conduct a structural analysis to identify how to arrange the environment differently Teach strategies to manage anger and frustration that all people encounter in day-to-day life • Teach Autistic people to self-advocate when they are being treated unfairly
Decision-Making about Interventions	• Intervening with the goal of “masking” Autistic features/traits or teaching Autistic people to “code-switch” when this is not their goal • Identifying intervention goals based only on the practitioner’s experience and/or caregiver preference • Failing to collect sufficient data on individual affect or individual intervention acceptability • Assuming that crying or other protest-related behavior is a “problem behavior” rather than a protest and failing to consider the reason for the protest • Failing to respect protests or refusal to engage in aversive activities • Assuming that mental health concerns are part of autism diagnosis rather than an area to target for support • Ignoring people’s choices on their chosen community (e.g., completing social skills programs with people with whom Autistic people do not want a social relationship)	• Teach self-advocacy skills from an early age and throughout lifespan (including advocating for priorities and preferences on intervention goals) (see Leadbitter et al., 2021) • Reduce coercive interactions during the teaching process by prioritizing assent-based interventions and least-restrictive environments • Identify the individual’s preferred targets for change during the instructional period and across long term goals • Promote collaborative and service user-led planning and intervention selection • Work in a cross-disciplinary way to identify additional supports that can help Autistic people achieve their preferred goals (e.g., yoga balls as alternative seating when desired) (Kunze & Machalicek, 2022; Slim & Reuter-Yuill, 2021) • Increase use of within stimulus prompts and/or prompting methods that are not intrusive • Adjust relevant features of intervention when a learner consistently refuses to assent or withdraws assent (Breaux & Smith, 2023) • Provide individually-identified support to Autistic adults (i.e., providing support across the lifespan) and consider the behavior analysts’ scope of competence and continuing education specific to adult services (Bahry et al., 2023) • Provide access to mental health services (Chan & Doran, 2024) • Providing opportunities for self-advocacy and self-agency in social contexts
Restrictive Environments and Procedures	• Assuming a more restrictive environment is required • Using restraint for any reason other than crisis involving safety • Failing to define safety crisis conditions that are likely to result in restraint • Using restraint until the individual is calm • Using restraint contingent on undesired behavior rather in a safety crisis	• Assume a less restrictive environment should be provided unless data show a more restrictive environment is necessary to address person-centered goals • Plan to fade the more restrictive environment starting from the onset of its application • If restraint is necessary, release from restraint the moment imminent danger has passed • Clearly define what constitutes a safety crisis risk. Conduct individualized risk assessments that incorporates safety risk in home, in clinic, on telehealth and in the community (see BACB Ethics Code, 2022 and UK-SBA Ethics Code, 2023 for guidance on safety and mitigating harm) • Educate staff regarding use of restraint only in safety crisis situations • Train a team member who is not involved in restraint to facilitate release as soon as the imminent danger has passed to reduce the likelihood restraint continues unnecessarily • Conduct discrimination training with staff regarding situations that constitute/do not constitute a safety crisis
Collaboration with Caregivers	• Assuming the caregiver speaks for the individual receiving services without considering the individual’s own competency to self-advocate • Assuming caregiver training is necessary only for those who seek this support • Focusing primarily on compliance training during caregiver training	• Ask the caregiver what the learner’s interests and priorities are so that goals are important ***to and for*** the learner (see Smith et al., 2006) • Provide caregiver education for caregivers of Autistic people that remediates existing ableist biases and does not perpetuate further ableist biases • Provide caregiver education for caregivers of Autistic individuals that teaches to (a) be informed consumers of behavior-analytic supports and services, (b) understand the benefits of behavior-analytic supports and services, (c) understand the ethical obligations of behavior analytic practitioners and recourse if a practitioner is not behaving ethically • Provide caregiver education for caregivers of neuro-majority children to cultivate inclusive environments • Providing caregiver education that is inclusive of Neurodivergent caregivers • Providing opportunities for networking and support systems between caregivers
Societal Changes	• Placing minimal emphasis on social validity • Using ABA only to make service user-level changes rather than societal changes • Failing to incorporate anti-ableism strategies in supervision and practice	• Feature Autistic representation with respect to decision-making, hire Autistic consultants and staff that specialize in behavior analytic and related services, include Autistics on organizational boards, pay Autistics to review intervention programs, pay or ask for and include input from Autistics when developing research agendas • Address all the ways ableism infiltrates practice with supervisees • Model anti-ableist actions (i.e., in workplace settings, in educational training settings by using anti-ableist language) • Call in/call out ableist actions of practitioners, parents, caregivers and community members • Advocate for legislation that supports the autonomy and self-determination of disabled people • Advocate for accessibility adjustments to sensory environments (e.g., autism inclusive hours in cinemas or shops that adjust the sensory environment; Chapman & Botha, 2023) • Plan for social validity measures to be embedded into empirical research

### How-to Guide

We offer the following practical suggestions informed by or in service of assent-based, compassionate, and socially valid practice. We posit that these specific topic areas address ableism within applied behavior analysis and provide one actionable recommendation including but not limited to examples of what may be current ableist practice and considerations for how to target this change in providing more anti-ableist services. We provide additional examples for each topic area in Table 1. This is not an exhaustive list and there is much still to consider. Where available, we have included references. Where references to the literature were not found, the recommendations were based on the personal and professional experience of the authors, consultation with Autistic people who are familiar with ABA-based services, or both.

### Working with Autistic People

#### Ableist Practice

Using infantilizing language or maintaining depictions of Autistic people as childlike when they are adolescents or adults.

#### Consider Targeting Practitioner Language Change

Prioritize and respect the Autistic person’s preferred terminology, use age and developmentally appropriate language in describing tasks and goals (Monk et al., 2022).

#### What Does that Mean for Practice?

When working with Autistic people who have a means of communicating their preferences, allow them to express whether they prefer person-first or identity-first language. Language should be individualized to meet the appropriate level of understanding and comprehension rather than based on practitioners' notions of what the Autistic person should, can, or cannot understand (Catala et al., 2021). Prioritize the person’s perspective as being the expert on their own experience and preferences, rather than non-autistic’s continuing to be representatives of autism (Akhtar et al., 2022; Bosco, 2023). For example, although we have used identity-first language throughout this manuscript, we would adapt our language whenever Autistic people prefer person-first language.

### Assessment

#### Ableist Practice

Conducting preference assessments to arrange contingencies but not to identify the Autistic individual’s preference for instructional programs.

#### Consider Targeting Practitioner’s Programmatic Decisions

Conduct assessments to identify skill strengths and interests (Woods & Estes, 2023).

#### What Does that Mean for Practice?

The behavior analyst should achieve a balance between goal development, skill acquisition programming and preferences of the Autistic person. This can be accomplished during instructional periods via methods like determining the sequence of programming that aligns with the individual’s preferences (e.g., providing a choice for working on reading skills or gross motor skills first; Deel et al., 2021). Further, the behavior analyst should seek to ensure that the assessment process is collaborative, striving to use neurodiversity-affirming assessments that center the Autistic person’s perspective while acknowledging the role of the environment (Pritchard-Rowe & Gibson, 2024).

### Selecting Service Goals

#### Ableist Practice

Setting goals that aim to change Autistic people so they behave more like the neuro-majority.

#### Consider Targeting the Autistic Person's Role in Selecting Service Goals

Respect preferences of Autistic people and include the person’s perspectives when developing behavioral target goals to ensure that goals are meaningful to them (Schwartz & Baer, 1991).

#### What Does that Mean for Practice?

When possible, during initial assessment stages, behavior analysts should seek to understand what goals the Autistic person would like to work toward and achieve in both the short- and long-term in addition to what the results of formal structured assessments may consider of importance to target. Determining what is of high priority to the individual can then be continuously programmed into services alongside other identified areas of support. Further, collecting feedback and ongoing evaluation from the individual on both the design and implementation of intervention/s should be sought to continue to provide socially valid and meaningful services that are in line with the individual’s preferences (Monahan et al., 2023).

### Decision-Making About Interventions

#### Ableist Practice

Behavior analysts selecting intervention procedures based on the organization’s procedural handbook (i.e., “this is the prompt hierarchy we use”), their own experience with and confidence in their ability to implement procedures with fidelity, or a list of evidence-based practices for a particular behavior target.

#### Consider Targeting Inclusion of All Interested Parties in Decision-making

Identifying options for the person receiving services and their caregivers (e.g., family members) to select from (Schuck et al., 2024).

#### What Does that Mean in Practice?

Survey results have emphasized prioritizing the individual receiving services and their family as the two most important perspectives when selecting service goals and procedures (Chazin et al., 2024). In some cases, interventions that prioritize following the learner’s lead may be favored (Schuck et al., 2024). In addition, the behavior analyst should take into consideration the results of a comprehensive assessment that includes gathering information about the Autistic person’s preferences, priorities, culture, and the family’s priorities and culture, when generating a range of options for instructional and intervention procedures. The behavior analyst should aim to be inclusive of the individual’s culture from the intake; this might include ensuring initial onboarding interviews are culturally informed (Jimenez-Gomez & Beaulieu, 2022), providing translation services and support, and de-centering the behavior analyst’s own notion on what should be prioritized. Including culturally relevant stimuli in activity choices and supporting any accessible communication forms rather than just spoken language are additional ways of centering the Autistic learner and promoting culturally relevant instruction and support (see Schuck et al., 2024).

### Restrictive Environments and Procedures

#### Ableist Practice

Assuming a more restrictive environment is required.

#### Consider Targeting Least Restrictive Practices

Assume a less restrictive environment should be provided unless data show a more restrictive environment is necessary to address person-centered goals.

#### What Does that Mean for Practice?

Completed safety assessments and risk assessments should determine the level (if any) of the restrictive environment that is needed for each individual (Ducarre, 2023). Reviewing these assessments should be embedded as part of practitioner training along with continuing to obtain assent from individual service users. Further, as part of practitioner training, safety and risk assessments should define what constitutes a safety crisis and a plan for fading any restrictive environments as soon as imminent danger has passed. Risk assessment and safety records should be individualized and not interchangeable across people. Records of engaging in restrictive environments and procedures should be regularly reviewed after and practitioners should continue to assess the need for restrictive practices per each individual.

### Collaboration with Caregivers

#### Ableist Practice

Assuming the caregiver speaks for the individual.

#### Consider Practitioners Collaborating with Caregivers

Prioritize the interests and needs of the individual in conjunction with caregiver interests.

#### What Does that Mean for Practice?

Obtaining assent and consent from the individual as well as the caregivers when selecting service goals. Work with caregivers to help the individual work toward becoming more autonomous in their decision-making. Although medical decision-making for minors is complex involving many legal and ethical considerations (Späth & Jongsma, 2020), assent and consent of the individual as they develop their autonomy should be considered throughout the development of service goals and programming (Baiden et al., 2024; Linnehan et al., 2023). For example, sometimes a caregiver expresses interest in their child learning to pick out “appropriate clothes” but the child shows a strong preference for particular clothes that are not “appropriate” according to the caregivers. In such cases, try to determine the difference between the caregiver’s and child’s preferences. Does the child prefer clothes that don’t constrict movement (e.g., tank tops and shorts), don’t have tags (consider sensory sensitivities), or have favorite animated characters on them? After narrowing the hypothesis regarding the reason for the child’s preference, work to negotiate with the caregiver on the child’s behalf for selecting the child’s preferred clothes.

### Societal Changes

#### Ableist Practice

Failing to incorporate anti-ableism strategies in supervision and practice.

#### Consider Targeting Societal Change within Practitioner Training Programs

Address the ways ableism infiltrates practices with supervisees.

#### What Does that Mean for Practice?

Plan for supervisee training on anti-ableist strategies, including holding space to consider and reflect on anti-ableist strategies as part of practitioner training and onboarding, and incorporating Autistic person’s perspectives (Baiden et al., 2024; Bent et al., 2024) throughout.

We would be remiss if we did not acknowledge the complexity of addressing ableism in the context of serving the heterogeneous Autistic population that includes a range of strong cognitive skills to severe cognitive impairments and people with strong vocal language skills to non-speaking people. Finally, we would be neglectful if we also did not acknowledge the additional challenges that Autistic adults, including non-speaking and minimally vocal individuals, face with regard to accessing validated assessment and support for mental health needs (Camm-Crosbie et al., 2019). When a person requires supports, particularly early and life-long substantial support, tensions arise between behavior targets that caring family members and practitioners believe are important ***for*** the individual and what is important ***to*** the individual (Callicott, 2003). Yet, these two perspectives need not be mutually exclusive. Behavior analysts who design support programs can adopt a supported decision-making process (Lill et al., 2021) to plan for instruction and support that includes targets that are both important ***to*** and ***for*** the individual.

## Summary

This paper has described some preliminary actions behavior analysts can take to dismantle ableism in practice. The suggestions and situations are informed by both experiences and observations in behavior analysis practice and the literature. However, these are merely preliminary actions, and the authors acknowledge that the contents here are incomplete. Where applicable, references to the existing literature have been included, yet we recognize that much of this work remains at a conceptual or discursive phase, with empirical action in its early phases. In particular, further work related to intersectionality of marginalizing identities such as race, culture, or identity with disability present additional complexities requiring culturally responsive approaches to assessment, intervention, and goal section and implementation are encouraged (Catrone et al., 2023; Jimenez‐Gomez & Beaulieu, 2022; Mathur & Rodriguez, 2022). Historically, systemic ableism has limited all but a tiny fraction of Autistic people’s access to advanced degrees in higher education that prepare one to conduct empirical research. Therefore the majority of their contributions to the literature in ABA have been in the form of blog posts, podcasts, and other forms of social media in which they describe their lived experience. Fortunately that is changing. Nonetheless, all these sources as well as qualitative and other published research by Autistic people that identify key themes in Autistic people’s experience in an ableist society (Bargiela et al., 2016; Santhanam & Hewitt, 2021) will enhance behavior analysts’ awareness of cultural differences between Neurodivergent and neuro-majority groups and facilitate altered perspectives of Autistic people. In turn, behavior analysts can employ the many tools at their disposal to move from awareness to actively providing anti-ableist, person-centered care (Choy-Brown, 2021). Although there are numerous further issues that merit exploration with respect to dismantling ableism, we present this paper as a beginner’s guide to support practitioners in actively challenging ableism within ABA practice.

## Data Availability

No data were collected or analyzed for this paper; there are no data available.

## Footnotes

1 For more, see https://rehabpsych.org/lee-meyerson

2 The topic of ableism in applied behavior analysis has been raised by the Neurodivergent community. Autism is a form of neurodivergence and is the focus of this paper. In the Autism community, many self-advocates and their allies prefer terminology such as “Autistic,” “Autistic person,” or “Autistic individual” because autism is an important part of their identity—the same way one refers to Black people or D/deaf people. Simultaneously, we recognize that some people prefer person-first language and still others are not able to convey whether they have a preference and what that preference is. With respect for our Autistic colleagues and friends, we use identity-first language in this document when discussing issues specific to Autistic people. Throughout this document “Autistic” is capitalized to reflect the culture of the Autism community (Pittsburgh Center for Autistic Advocacy, 2018), whereas autism, referring to the diagnostic category, is not capitalized.

3 We use stigma here to refer to misconceptions and negative stereotypes.

4 Those who are not like us are considered the “other” and are members of the “outgroup” (Kitzinger & Wilkinson, 1996). See Calma-Roddin (2024) for elaboration on in-group and out-group dynamics and teaching these concepts in psychology and education contexts.
